# Synovial sarcoma with relevant immunocytochemistry and special emphasis on the monophasic fibrous variant

**DOI:** 10.4103/0970-9371.70736

**Published:** 2010-04

**Authors:** Radhika Kottu, Aruna K Prayaga

**Affiliations:** Department of Pathology, Nizam’s Institute of Medical Sciences, Punjagutta, Hyderabad - 500 082, Andhra Pradesh, India

**Keywords:** Immunocytochemistry, synovial sarcoma, fine needle aspiration cytology

## Abstract

**Background:**

Monophasic fibrous synovial sarcoma (SS) is the most common variant of SS. Only a few cytological studies are available on this entity. Bcl-2 protein expression has been described as a characteristic marker of SS and is useful for its differentiation from other sarcomas. Cytokeratin and CD99 are also used in detecting SS.

**Aims:**

To evaluate synovial sarcoma and its variants cytomorphologically.

**Materials and Methods:**

During a period of 10 years 7 months, i.e. from January 1998 to July 2008, 12 cytologic specimens diagnosed as synovial sarcoma were reviewed. Ten cases were diagnosed as SS on aspiration alone but two cases required ancillary technique i.e., immunocytochemistry staining with bcl-2 and cytokeratin. The smears were stained with Papanicolaou and May-Grünwald-Giemsa stains.

**Results:**

All cytologic specimens in our study had similar appearance. Most smears were highly cellular and were made up of densely packed tri-dimensional groups and singly scattered round to oval cells. Cellular monomorphism and vascular channels within the cell groups were the remarkable findings. Only one case showed cytologic evidence of epithelial differentiation. Bcl-2, cytokeratin, CD99 positivity was seen on immunohistochemistry staining. Results were categorized according to age, sex and morphologic variants.

**Conclusions:**

Although cytomorphologic features of synovial sarcomas are characteristic enough to permit its recognition, clinical correlation is necessary for accurate diagnosis. Monophasic variant is the most common entity observed in the present study.

## Introduction

Synovial sarcoma (SS) is a mesenchymal spindle cell tumor which displays variable epithelial differentiation including glandular formation and has a specific chromosomal translocation t (×:18) (p11:q11)[1] and accounts for 5–10% of all soft tissue sarcomas. Age of incidence varies from birth to the maximum age of 89 years. It is commonly seen among adults with male preponderance. The tumor is of unknown histogenesis and is unrelated to synovium.[2] It arises most commonly from the deep soft tissues of extremities. Four morphologic variants have been described: classic biphasic type, monophasic fibrous type, monophasic epithelial type and poorly differentiated (round cell) type.

Monophasic fibrous SS is the most common variant of SS. Only a few cytologic studies are available on this entity. Bcl-2 protein expression has been described as a characteristic marker of synovial sarcoma. Cytokeratin is also used in detecting this soft tissue tumor.

## Materials and Methods

Twelve cytologic cases of SS were studied in a retrospective analysis over a period of 10 years 7 months, i.e. from January 1998 to July 2008. All specimens were obtained from fine needle aspiration cytology (FNAC) using 23–25 gauge needle. Wet and air dried smears were prepared and stained subsequently with Papanicolaou and May-Grünwald-Giemsa stains, respectively. Ten cases were diagnosed on FNAC alone. Two cases required immunocytochemistry in view of the poor cellularity and staining for bcl-2 and cytokeratin were done. Of the total twelve cases, histopathologic correlation was available only for five cases. Histopathology ascertained the cytologic diagnosis in these five cases.

## Results

Clinicopathologic features are summarized in Tables 1 and 2. There were seven males and five females. Age ranged from 12 to 65 years with a mean age of 32.9 years. Thigh was the primary site in seven cases. Single case was obtained from gluteal region, shoulder, arm, calf and knee joint. Recurrent tumor was noted in two cases and metastases was observed in three cases. Metastases were seen in the lung followed by lymph node. Monophasic SS accounted for 83% of the cases and biphasic variant 17%. The initial cytologic diagnosis of the monophasic synovial sarcoma was a malignant spindle cell tumor possibly of synovial sarcoma. Histologic correlation was available in five cases. Mass lesion was the presenting symptom in majority of the patients.

**Table 1:** Clinical features of patients

Patient no.	Age (Yrs)	Gender	Primary site	Cytology site	Cytologic material
1	21	M	Gluteal region	Primary	FNAC
2	12	F	Thigh	Recurrence	FNAC
3	55	M	Thigh	Primary	FNAC
4	30	M	Shoulder	Primary	FNAC
5	33	M	Thigh	Primary	FNAC
6	35	M	Arm	Primary	FNAC
7	49	M	Thigh	Lymphnode mets	FNAC
8	17	F	Thigh	Recurrence	FNAC
9	23	M	Calf	Primary	FNAC
10	30	F	Thigh	Pulmonary mets	FNAC
11	25	F	Knee joint	Pulmonary mets	FNAC
12	65	F	Thigh	Primary	FNAC

**Table 2:** Pathologic features of tumors

Cytodiagnosis (site)	ICC	Histo Diagnosis	IHC
SS (Primary)	–	Monophasic	CytoK, S100 protein, CD34
Biphasic SS (Primary)	–	Biphasic	–
SS (Primary)	–	Monophasic	–
SS (Metastases)	–	Monophasic	–
Biphasic SS (Metastases)	–	Biphasic	–

ICC - Immunocytochemistry, IHC - Immunohistochemistry

The monophasic sub type showed spindle cell sarcoma with hemangiopericytoma like foci and the biphasic type contained epithelial-like glands and nests. Presence of mast cells is also an important differential diagnostic feature of synovial sarcoma. Our results showed the same in three out of five cases. Depending on the availability of the markers, cytokeratin, S100 protein and CD34 were done. The S100 protein was done to exclude nerve sheath tumor. Cytokeratin and CD34 were positive and S100 protein was negative. The immunophenotype tends toward schwannian differentiation when S100 positivity is demonstrated (only in 50% of the cases), but is more commonly positive for LEU-7, collagen type 4 and myelin basic protein.

## Cytologic findings

**Figure 1:**
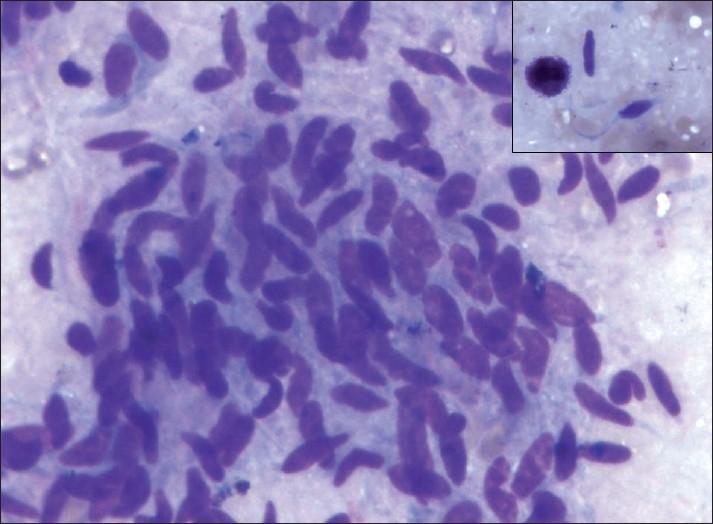
Spindle cells forming whorls MGG (40×10) (inset shows mast cell)

**Figure 2:**
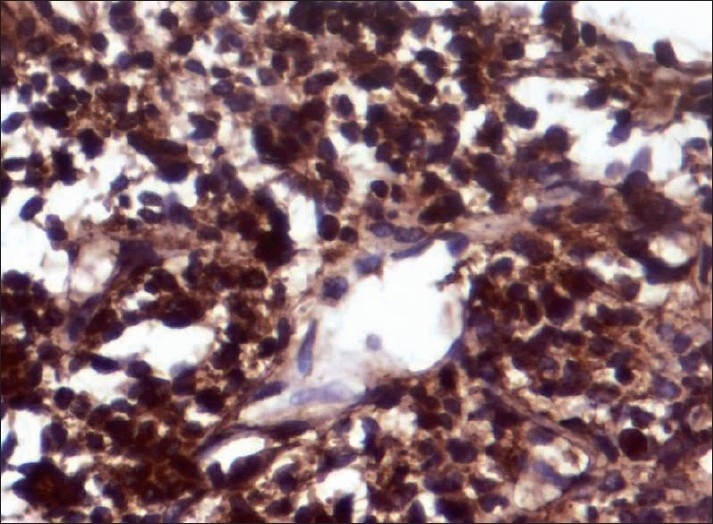
Cytokeratin positivity on immunocytochemistry

**Figure 3:**
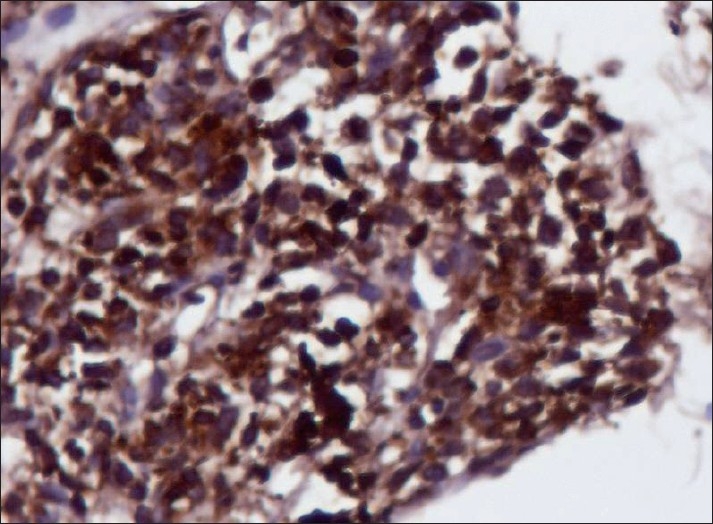
Bcl-2 positivity on immunocytochemistry

All smears showed similar morphology. The smears were highly cellular with clusters and dispersed cell population. There were abundant single cells with naked nuclei. Cells were elongated, spindle to oval in shape and had uni and bipolar cytoplasmic processes. Nuclei were pleomorphic with hyperchromasia. There was presence of mast cells with infrequent mitoses and occasional calcifications. Myxoid matrix was seen stained purple to magenta color on May-Grünwald-Giemsa. Cell clusters were showing whorls and pericytic patterns in some and micro acini in a few cases [Figure 1]. Clusters of round epithelial cells with vesicular nucleus and inconspicuous nucleoli were also observed. Both epithelial and mesenchymal elements were seen in two cases of biphasic variant of synovial sarcoma. Single case showed metachromatic stroma in the background. Immunocytochemistry was required in two cases. Bcl-2 showed strong positivity and cytokeratin was less intensely positive. Both patients were male, aged 55 and 33 years, respectively, presented a thigh lesion (Table 1, Patient no. 3 and 5). These patients’ smears were less cellular and showed spindle cells arranged in whorls and single scattered forms along with mast cells. A malignant spindle cell tumor was favored on cytology keeping synovial sarcoma as a possibility correlating with clinical features, i.e. thigh lesion. Immunocytochemistry, i.e. cytokeratin and bcl-2 were advocated which showed positivity [Figures 2 and 3] and final impression of synovial sarcoma was made. bcl-2 positivity was restricted to spindle cells in biphasic and monophasic synovial sarcomas. The question of differential diagnosis of monophasic fibrous synovial sarcoma with follicular lymphoma hence did not arise. However, in poorly differentiated synovial sarcomas this is important – in that also morphology (follicles) helps. As the patients did not turn up to the surgical department, biopsy was not done in both the cases.

## Discussion

FNAC plays a major role in diagnosing pre-operative mesenchymal lesions and distinguishes both benign and malignant soft tissue lesions. There are no major studies from India on synovial sarcoma alone emphasizing the role of FNAC in diagnosing the same except for a few case reports. Kumar *et al*.[3] and Dey *et al*.[4] published their studies in diagnosing soft tissue tumors from India. Different cytologic series report positive and negative predictive values of 85–100% and 94–100%, respectively.[3–5] Biphasic nature is the most distinctive histologic feature of synovial sarcoma.[6] A specific cytologic diagnosis is possible when there is distinct biphasic pattern. In many biphasic tumors cytology fails to sample the epithelial component preventing a specific diagnosis. Monophasic fibrous variant is more common among synovial sarcomas though the biphasic variant is the first described entity on cytology.[7,8] Many biphasic tumors lose their epithelial component either at recurrence or metastases.[2] Hence, it is necessary to emphasize the cytologic features of synovial sarcoma other than epithelial morphology. High cellularity, cellular anaplasia, foci of necrosis and a high mitotic index indicates aggressive behaviour of the lesion.[9] In addition to these features, obligatory clinico radiologic correlations are necessary for a reliable diagnosis of malignancy. Synovial sarcoma is a high grade neoplasm with remarkably uniform nuclei and evenly distributed chromatin. Necrosis is uncommon in synovial sarcoma but encountered in recurrent or metastatic diseases or cases with therapeutic irradiation.[10] Present study does not show any evidence of necrosis. Mitoses are uncommon in synovial sarcoma except for poorly differentiated variants.[2] Infrequent mitoses were noted in our study. Monophasic synovial sarcoma is a highly cellular tumor almost devoid of stroma. Only a single case showed such evidence in the present study. Calcification, which is usually described with synovial sarcoma was found infrequently. Monophasic synovial sarcoma is prevalent in adolescents and young adults. Viguer *et al*.[9] in his study showed the prevalence in adolescents and young adults. Present study also shows the same. Male preponderance is observed in the present study whereas Viguer *et al*. showed female preponderance in the eleven cytologic specimens studied. The primary site is thigh in majority of the cases, which is similar with the present study [Table 1]. Recurrent tumor were common after primary tumor according to Viguer *et al*., but our experience shows that metastatic tumor were common after primary tumor. All cytologic specimens were obtained from fine needle aspiration by Viguer *et al*., except for two cases obtained from tumoral fluid. Present study does not show any evidence of cystic areas even in a single case, and shows monophasic fibrous variant as the most common on cytology and on histopathologic correlation as well [Table 2]. This study is also supported by the study of Viguer *et al*. stating that monophasic variant is the most common type of all morphologic variants of SS.

SS has to be differentiated from two broad based categories of lesions:

1. Mesenchymal lesions with uniform spindle to round cell morphology
2. Tumors with epithelioid cell morphology

But the diagnostic difficulties are commonly encountered with hemangiopericytoma and fibrosarcoma.[11] Hemangiopericytic pattern is more commonly seen in hemangiopericytoma compared to synovial sarcoma. Such pattern was noticed in a single case in the present study. We could differentiate it from the presence of other cytologic features favouring synovial sarcoma. It has been proved that immunohistochemistry is crucial for the definite diagnosis of synovial sarcoma. Though keratin comes positive in all biphasic tumors and in many of the monophasic fibrous types according to Ackerman *et al*., it cannot be considered critical in the cytologic diagnosis of synovial sarcoma. Bcl-2 protein shows intense cytoplasmic positivity but has to be differentiated from bcl-2 positive lymphomas.[8] Thirty percent of synovial sarcomas show S100 protein positivity but one has to keep in mind malignant peripheral nerve sheath tumor in ruling out as a possibility.[10] Sarcomatoid mesothelioma can be excluded by doing calretinin and HBME1. In difficult cases, interphase fluorescent insitu hybridization aids in diagnosing synovial sarcomas.[12] Cytogenetic analysis can be performed on FNAC specimens from soft tissue sarcomas and may be a useful diagnostic aid in difficult cases. However, when cell block material is available for immunohistochemistry, the majority of sarcoma subtypes with specific cytogenetic profiles can be successfully classified with or without cytogenetic analysis.[13]

Mohite *et al*.[14] in their publication on a single case report of synovial sarcoma stated that fine needle aspiration is less accurate than other techniques for the diagnosis of soft tissue tumors and that cytogenetic analysis is an important diagnostic tool. However, there are studies from India and abroad emphasizing the utility of fine needle aspiration cytology in diagnosing soft tissue tumors.

In the hands of experienced cytopathologists FNAC in conjunction with ancillary techniques has a diagnostic accuracy approaching 95% for the diagnosis of soft tissue sarcomas.[15]

Domanski[16] stated that the successful cytological evaluation of soft tissue lesions requires the application of strict, reproducible morphological criteria in the context of the clinical findings as well as ancillary techniques.

Although cytomorphologic features of synovial sarcoma are characteristic enough to permit its recognition, clinical correlation is necessary for its correct identification.
